# Efficacy and safety of ultrasound-guided pulsed radiofrequency in the treatment of the ophthalmic branch of postherpetic trigeminal neuralgia

**DOI:** 10.3389/fneur.2024.1398696

**Published:** 2024-05-28

**Authors:** Fubo Li, Gege Gong, Yue Zhang, Cehua Ou

**Affiliations:** ^1^Department of Pain Management, The Affiliated Hospital, Southwest Medical University, Luzhou, China; ^2^Department of Physical Diagnosis, The Affiliated Hospital, Southwest Medical University, Luzhou, China

**Keywords:** herpes zoster, pulsed radiofrequency, trigeminal neuralgia, ultrasound guidance, nerve block

## Abstract

**Objective:**

To investigate the efficacy and safety of ultrasound-guided pulsed radiofrequency (PRF) targeting the supraorbital nerve for treating the ophthalmic branch of postherpetic trigeminal neuralgia.

**Methods:**

A retrospective cohort study was conducted involving patients who presented at the Department of Pain, Affiliated Hospital of Southwest Medical University from January 2015 to January 2022. The patients were diagnosed with the first branch of postherpetic trigeminal neuralgia. In total, 63 patients were included based on the inclusion and exclusion criteria. The patients were divided into the following two groups based on the treatment method used: the nerve block (NB) group (*n* = 32) and the PRF + NB group (radiofrequency group, *n* = 31). The visual analog scale (VAS) score, Pittsburgh Sleep Quality Index (PSQI) score, and pregabalin dose were compared between the two groups before treatment, 1 week after the procedure, and 1, 3, and 6 months post-procedure, and the complications, such as local infection, local hematoma, and decreased visual acuity, were monitored post-treatment.

**Results:**

No significant difference was found in terms of pretreatment age, sex, course of disease, preoperative VAS score, preoperative PSQI score, and preoperative pregabalin dose between the two groups (*P* > 0.05). The postoperative VAS score, PSQI score, and pregabalin dose were significantly decreased in both groups. Furthermore, significant differences were found between the two groups at each preoperative and postoperative time point (*P* < 0.05). The VAS score was lower in the radiofrequency group than in the NB group at 1, 3, and 6 months, and the difference was statistically significant (*P* < 0.05). The PSQI score was lower in the radiofrequency group than in the NB group at 1 week, 1, 3, and 6 months post-procedure, and the difference was statistically significant (*P* < 0.05). The dose of pregabalin was lower in the radiofrequency group than in the NB group at 1 week, 1, 3, and 6 months post-procedure, and the difference was statistically significant at 3 and 6 months (*P* < 0.05). After 6 months of treatment, the excellent rate of VAS score in the radiofrequency group was 70.96%, and the overall effective rate was 90.32%, which was higher than that in the NB group. The difference in the efficacy was statistically significant (*P* < 0.05).

**Conclusion:**

PRF targeting the supraorbital nerve can effectively control the pain in the first branch of the trigeminal nerve after herpes, enhance sleep quality, and reduce the dose of pregabalin. Thus, this study shows that PRF is safe under ultrasound guidance and is worthy of clinical application.

## 1 Introduction

Postherpetic trigeminal neuralgia is a condition in which facial skin herpes arises due to varicella-zoster virus (VZV) infection of the trigeminal ganglion or branches. Facial pain stops or reappears after herpes scab. This type of facial pain belongs to one of the special types of postherpetic neuralgia (PHN) and often presents with burning, shock-like, and needle-like pain. Severe pain can often last for many months or even years (1). Pregabalin, oxcarbazepine, and other first-line antineuralgia drug treatments are commonly used; however, increasing the drug dose may lead to dizziness, drowsiness, and other drug-related side effects, which limit the usage of drugs. The nerve block (NB) is effective in treating PHN; however, it requires repeated multiple injections of analgesic solutions and is, therefore, often not ideal. Because analgesic solution contains steroid hormones, repeated injections can increase the risk of infection in patients with diabetes and individuals with low immunity, limiting the use of NBs to some extent (2).

Recently, pulsed radiofrequency (PRF) has been used to generate high voltage around nerve tissue via pulsed current, regulate pain afferent pathway nerves, reduce inflammatory mediators around damaged nerves, and activate descending inhibitory pathways of pain to produce analgesic effects (3). PRF exhibits no destructive effect on nerve fibers, can reduce pain, and also exhibits less hypoesthesia, soreness, burning pain, and motor nerve injury post-treatment. It should be gradually applied to patients with neuralgia (3). Present studies on postherpetic trigeminal neuralgia have reported that semilunar ganglion nerve block of the trigeminal nerve, ozone injection, PRF, and puncture therapy can be performed under the guidance of computed tomography (CT) and digital subtraction angiography (DSA) (4). However, these methods involve deeper regions of the brain and have the disadvantages of oculocardiac reflex, intracranial infection, ophthalmic nerve injury, and large radiation during puncture and treatment, making them difficult to perform widely (1, 5). Furthermore, very few relevant studies on the use of PRF for targeting peripheral nerves in postherpetic trigeminal neuralgia have been reported. Whether the radiofrequency of trigeminal nerve branches can compensate for this defect and maintain efficacy is not known. Recently, with the development of musculoskeletal ultrasound, accurate PRF targeting peripheral nerves is not possible (6). Therefore, in this retrospective study, we analyzed the efficacy and safety of ultrasound-guided PRF targeting the supraorbital nerve for treating the ophthalmic branch of postherpetic trigeminal neuralgia.

## 2 Materials and methods

### 2.1 Clinical data

**Figure 1:**
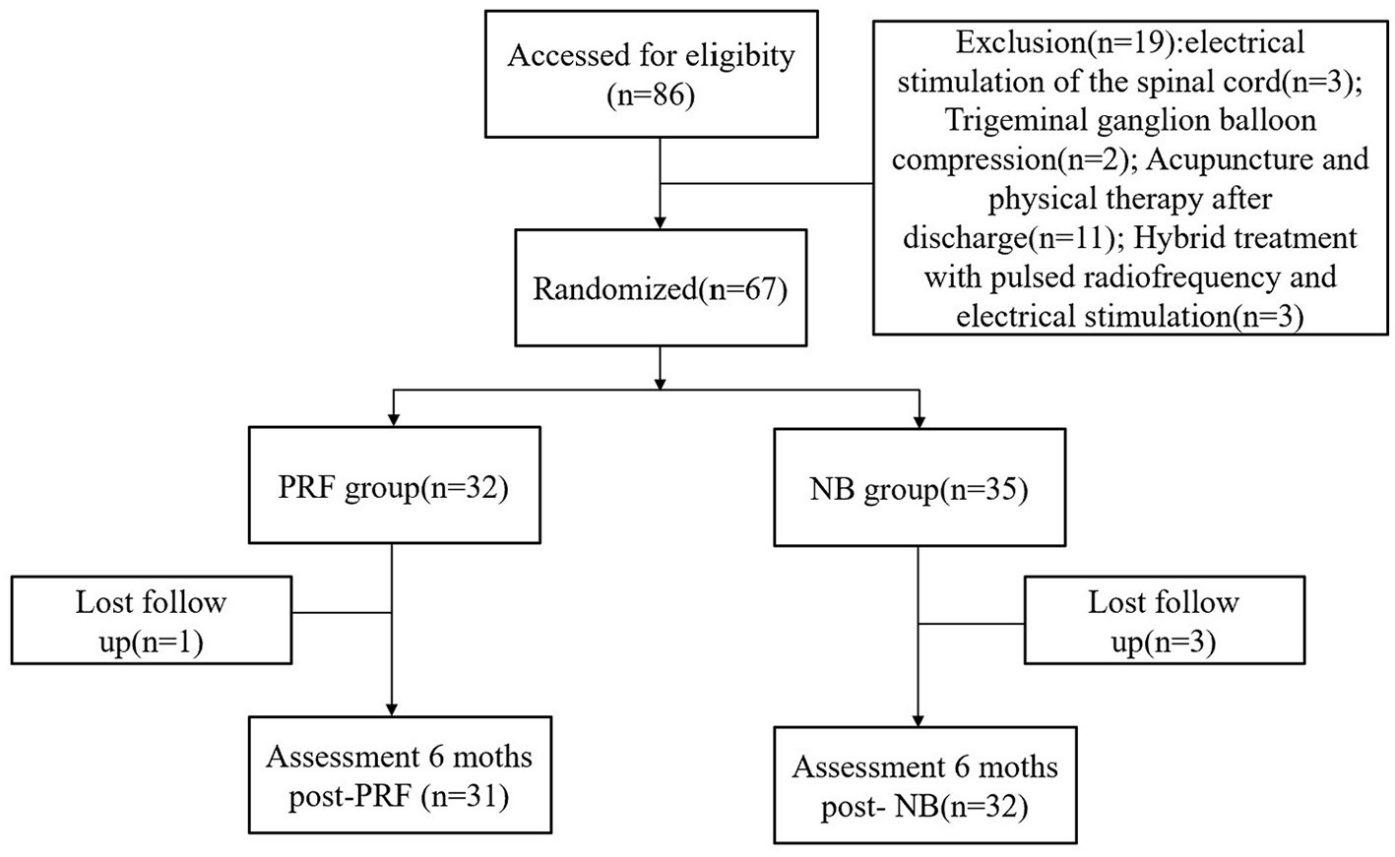
Study population and follow-up flowchart.

This study was retrospective in nature, and the Clinical Trial Ethics Committee of the Affiliated Hospital of Southwest Medical University agreed to the study protocol and approved the application form for waiving the signed consent form(registration number: KY2023245). The study meets the relevant requirements of the Declaration of Helsinki of the World Medical Association. The data included in the analysis were de-identified. In total, 86 patients with the ophthalmic branch of postherpetic trigeminal neuralgia who were diagnosed and treated at the Department of Pain, Affiliated Hospital of Southwest Medical University from January 2015 to January 2023, were selected, and 63 patients (Figure 1) were included based on the inclusion criteria and exclusion criteria. There was no sex limitation, the patients' age ranged from 42 to 86 years (63.58 ± 11.87), and the duration of the treatment was 30–7,200 days (206.04 ± 917.89). The patients were divided into the following two groups based on the treatment method: the nerve block group (NB group) and the PRF-combined NB group, hereinafter referred to as the radiofrequency combined NB group (PRF group). In total, 32 patients were present in the NB group, and 31 patients were present in the PRF group.

The inclusion criteria were as follows: (1) the area involving the nerve was the first branch of the trigeminal nerve; (2) the diagnosis of the patient was PHN (2); (3) no significant pain relief was achieved after nonsurgical treatment (including drugs and traditional therapy); and (4) the visual analog scale (VAS) score was ≥ 4.

The exclusion criteria were as follows: (1) patients with local ulcerated infection; (2) those with severe cardiopulmonary failure; (3) those with mental disorders; (4) those with coagulopathy; (5) those in which other treatments, such as spinal cord stimulation, trigeminal semilunar ganglion balloon compression, and traditional Chinese medicine, were used during treatment; (6) those who received other treatment options after discharge; and (7) those lost to follow-up or with incomplete data.

### 2.2 Treatment methods

The experimental set-up included radiofrequency equipment and drug ultrasound model Terason t3000TM ultrasound system (manufactured by Teratech, Burllington, MA01803, USA). A high-frequency linear array probe was used for probe selection. The radiofrequency thermostat (model R-2000BA1, Beijing Beiqi Medical Technology Co., Ltd.) and radiofrequency electrode trocar (model 20G, Innoman Medical Technology Co., Ltd.) were used. As a part of the experimental protocol, lidocaine hydrochloride injection (Double-Crane Pharmaceutical Co Ltd), dexamethasone sodium phosphate injection (Dexamethasone, Zhengzhou, Zhuofeng Pharmaceutical Co. Ltd), and mecobalamin injection (Mecobalamin, Mecobalamin) were also used. Both groups were treated with ultrasound-guided supraorbital nerve therapy.

**Figure 2:**
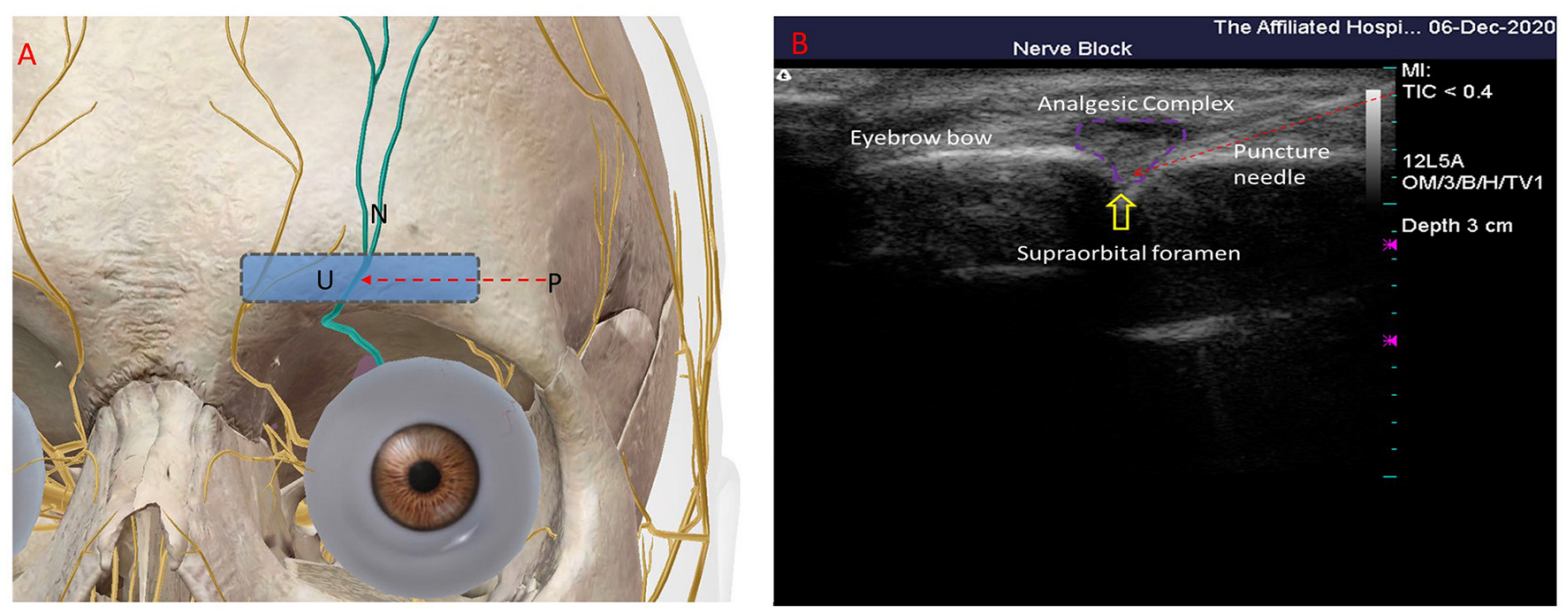
Ultrasound-guided supraorbital nerve treatment. **(A)** Schematic of ultrasound probe placement; P, puncture needle; U, ultrasound line array probes; N, supraorbital nerve. **(B)** The signal interrupted on the arch of the eyebrow is the supraorbital notch (indicated by the yellow arrow in the figure); the puncture needle is a strongly echogenic acoustic shadow above the red arrow, oriented as indicated by the red arrow in the figure; the hypoechoic surrounding the supraorbital notch is the analgesic complex fluid—the area surrounded by the purple line.

In the NB group, the patients were placed in the supine position with a padded pillow below the head. The neck was slightly flexed, and the frontofacial eyebrow arch was located via ultrasound. Continuous hyperechoic cortical acoustic shadows, i.e., the eyebrow arch, were observed. The ultrasound was moved caudally, and the hyperechogenicity showed interrupted notches, i.e., the supraorbital foramen or supraorbital notch (Figure 2). After routine disinfection and draping, local intradermal injection of 1% lidocaine anesthesia was administered. To perform the ultrasound-guided in-plane technique, a needle was subcutaneously punctured through the skin, avoiding the supraorbital artery, to the supraorbital foramen (or supraorbital notch). Then, 1 mL of the analgesic complex solution was injected (containing 40 mg of 2% lidocaine injection + 0.5 mg of mecobalamin injection + 5 mg of dexamethasone sodium phosphate injection + 10 mL of 0.9% sodium chloride in). After injecting, the patient was observed for 15 min and returned to the ward after confirming that there was no abnormality.

In the radiofrequency group, the localization was the same as that in the NB group. After routine disinfection and draping, 1% lidocaine was injected as the local anesthesia. The supraorbital artery was avoided under ultrasound guidance. After radiofrequency electrode trocar puncture into the supraorbital foramen (or supraorbital notch), frontoparietal tingling sensation was induced via electrical stimulation at 0.2–0.3 V and 50 Hz. A tingling sensation was induced in all patients. PRF selection parameters were as follows: 70 V, 2 Hz, 20 mS, 40°C, and 6 min and 70 V, 2 Hz, 20 mS, 42.0°C, and 6 min. Then, 1 mL of analgesic complex solution was injected at the end of surgery. After the surgery was complete, the patient was observed for 15 min and returned to the ward after confirming that there was no abnormality.

### 2.3 Efficacy evaluation methods

#### 2.3.1 Pain evaluation

Pain was evaluated using the VAS score, which was expressed as the intensity of pain from 0 to 10. A score of 0 indicated no pain, whereas a score of 10 indicated severe pain. The patients expressed the degree of pain based on their personal pain perception. Patients were assessed preoperatively and 1 week, 1 month, 3 months, and 6 months postoperatively.

#### 2.3.2 Sleep quality

The Pittsburgh Sleep Quality Index (PSQI) was used for sleep quality scoring, which consisted of 24 questions divided into seven categories (7). Each category had a score of 0–3 points; 0 points indicated no problem in sleeping whereas 3 points indicated difficulty in sleeping. The total score of each category accumulated, with a total score range of 0–2l. The higher the score, the worse the sleep quality.

#### 2.3.3 Pregabalin dose

Pregabalin dose was recorded at 6 months after surgery.

#### 2.3.4 Efficacy evaluation

The VAS weighted calculation method was used to evaluate the pain relief of patients at 6 months after surgery. Combined with the modified MacNab evaluation criteria, (8) the efficacy was divided into the following four levels: excellent, good, fair, and poor. The excellent effect was that the pain disappeared and the VAS score decreased by > 75%. The good effect was that the VAS score decreased by 51%−75%. The fair effect was that the VAS score decreased by 26%−50%, and the poor effect was that the VAS score decreased by ≤ 25%.

The response rate was calculated as follows: (excellent + good + fair)/total number of cases × 100%.

#### 2.3.5 Complications during surgical treatment

The patients were followed up for 6 months after surgery. Incidence of local infection, local hematoma, scalp numbness, abnormal vision, and other complications during and after PRF was recorded.

### 2.4 Statistical methods

Data was processed using the IBM SPSS 26.0 software, and GraphPad Prism9 was used for mapping the results. The data conforming to normal distribution are expressed as mean ± standard deviation (± *s*), and the differences between the two groups were compared using an independent sample *t*-test. The intragroup comparisons were performed using one-way ANOVA, and repeated comparisons were performed using Bonferroni. Enumeration data are presented as frequency and rate, and differences were compared using the χ^2^ test. Rank data were compared using the nonparametric rank-sum test. A *P* < 0.05 was considered statistically significant.

## 3 Results

### 3.1 Comparison of preoperative general conditions between the NB and PRF group

There was no significant difference in age, sex, course of disease, VAS and PSQI scores, and pregabalin dose between the two groups before surgery (*P* > 0.05) (Table 1).

**Table 1:** Comparison of preoperative general conditions between the two groups.

**Group**	**Number of cases**	**Age**	**Sex (male/female case)**	**Disease duration (Day, $\bar{x}$ ±*s*)**	**Preoperative VAS score (min, $\bar{x}$ ±*s*)**	**Preoperative PSQI score (min, $\bar{x}$ ±*s*)**	**Pregabalin (mg, $\bar{x}$ ±*s*)**
NB group	32	63.5 ± 12.1	21/11	107.1 ± 255.5	6.7 ± 0.7	16.4 ± 2.7	192.1 ± 68.5
PRF group	31	63.6 ± 11.7	18/13	308.0 ± 1,285.5	6.4 ± 0.8	16.3 ± 1.9	179.0 ± 76.6
*t* (χ^2^) value	-	−0.059	0.610	−0.867	1.676	0.189	0.719
*P*-value	-	0.953	0.544	0.389	0.099	0.851	0.475

VAS, visual analog scale; PSQI, Pittsburgh Sleep Quality Index; NB, nerve block; and PRF, pulsed radiofrequency.

### 3.2 Observations regarding the VAS score

**Figure 3:**
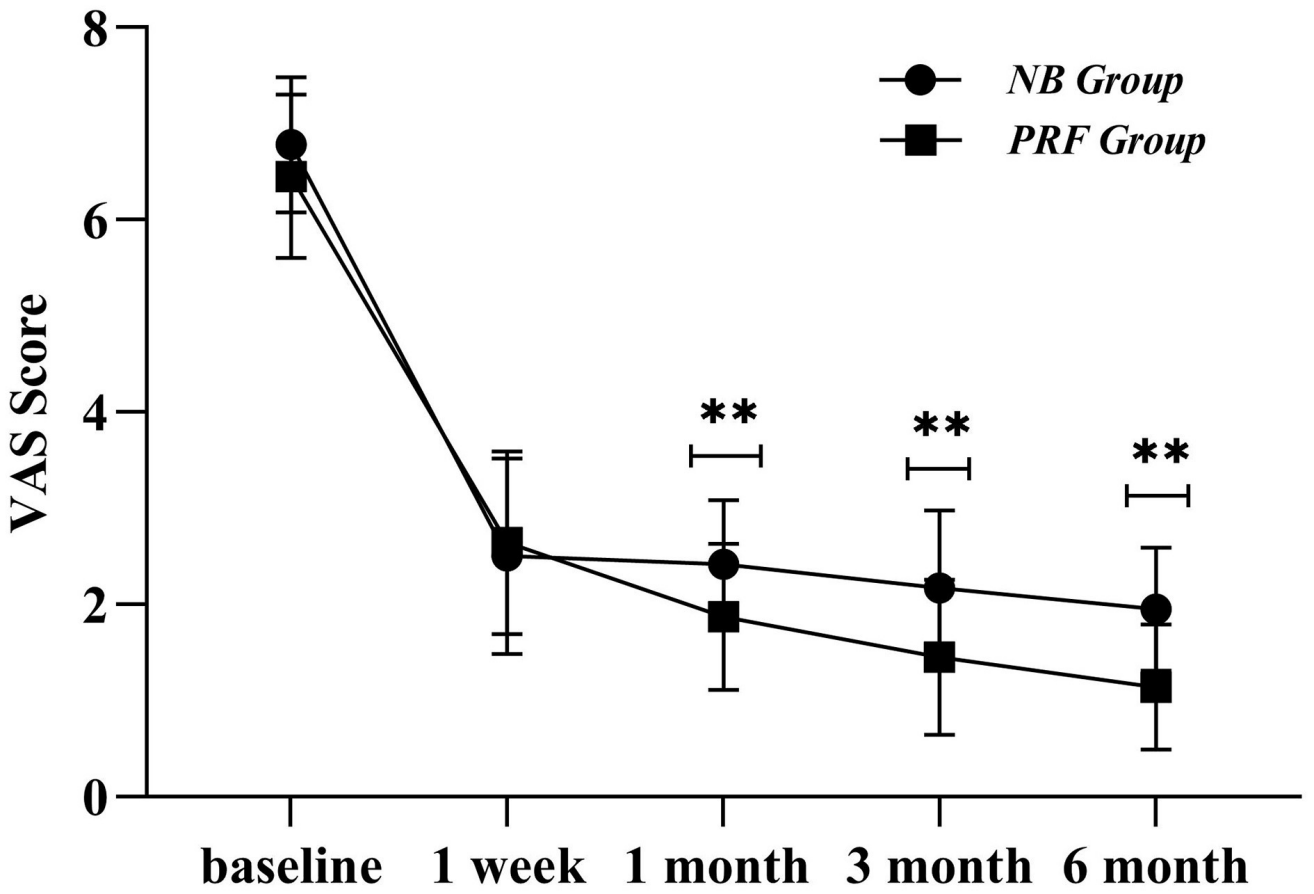
Postoperative VAS scores of the two groups of cases; **indicates *P* < 0.01.

The postoperative VAS scores of both PRF and NB groups were lower than the preoperative VAS scores at 1 week, 1 month, 3 months, and 6 months after surgery. The VAS scores of the PRF group gradually decreased from 1 to 6 months after surgery. The difference between the two groups at postoperative 1, 3, and 6 months was statistically significant (*P* < 0.01) (Figure 3), i.e., the VAS scores at a postoperative interval of 1 month indicated that the PRF group had better pain control (Figure 3).

### 3.3 Observations regarding the PSQI score

Compared with the preoperative conditions, significant differences were found in the general conditions in the NB group at postoperative intervals of 1 week, 1 month, 3 months, and 6 months (*P* < 0.05), indicating that the sleep quality was improved after the surgery. When compared within the groups at postoperative 1 week, 1 month, 3 months, and 6 months, the general conditions showed no statistically significant differences (*P* > 0.05). Compared with preoperative conditions, there were significant differences in the general conditions in the PRF group at postoperative intervals of 1 week, 1 month, 3 months, and 6 months (*P* < 0.05), indicating that the sleep quality was improved after the surgery.

**Figure 4:**
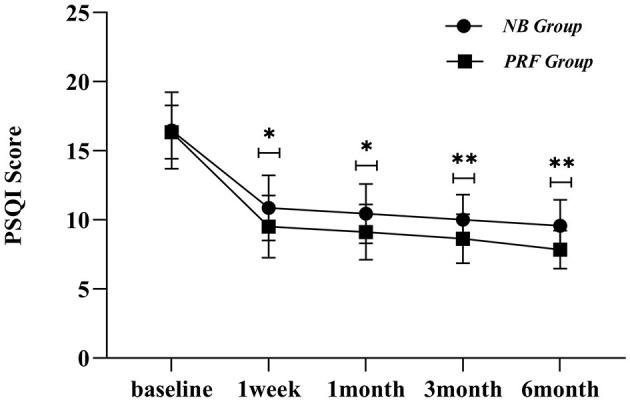
Postoperative PSQI scores in both groups; *indicates *P* < 0.05, **indicates *P* < 0.01.

The comparative PSQI scores between the PRF group and the NB group at postoperative intervals of 1 week, 1 month, 3 months, and 6 months are presented in Figure 4, and the differences were statistically significant (*P* < 0.05). The PSQI scores of the PRF group were lower than those of the NB group after the surgery, indicating that the sleep quality in the PRF group was better than that of the NB group.

### 3.4 Pregabalin dose

**Figure 5:**
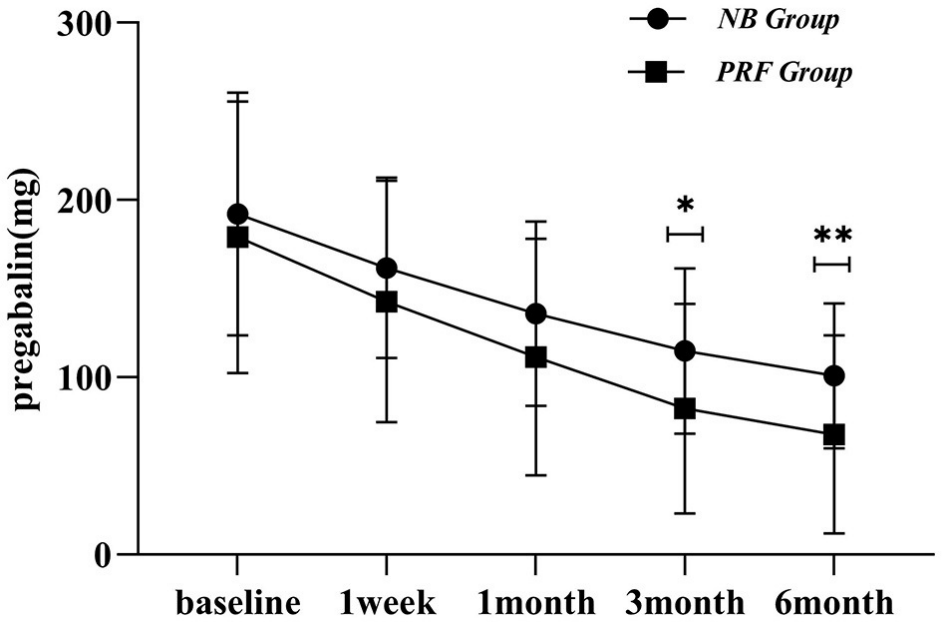
Comparison of postoperative oral pregabalin in the two groups, *indicates *P* < 0.05, **indicates *P* < 0.01.

Compared with the preoperative conditions, there were significant differences in the general conditions in the NB group at postoperative intervals of 1 week, 1 month, 3 months, and 6 months (*P* < 0.05), indicating that the dose of oral pregabalin was reduced after the surgery. When compared within the groups at postoperative intervals of 1 week, 1 month, and 3 months, the general conditions showed no statistically significant differences (*P* > 0.05). However, there were statistically significant differences between general conditions at postoperative 6 months and postoperative intervals of 1 week, 1 month, and 3 months (*P* < 0.05). Compared with the preoperative conditions, there were significant differences in the general conditions in the PRF group at postoperative intervals of 1 week, 1 month, 3 months, and 6 months (*P* < 0.05), indicating that the dose of oral pregabalin decreased after the surgery (Figure 5).

There were significant differences in pregabalin dose between the PRF and NB groups at postoperative intervals of 3 and 6 months (*P* < 0.05). The PRF group was administered fewer pregabalin doses after surgery than those administered in the NB group.

### 3.5 Efficacy evaluation of treatments in the two groups at a postoperative interval of 6 months

After a follow-up of 6 months, the effect in the PRF group was excellent compared with that in the NB group, with an overall response rate of 90.32%, whereas the overall response rate in the NB group was 81.25%. The nonparametric rank-sum test analysis of the response rate in the two groups showed a Z value of −2.055 and *P*-values of >0.040 and < 0.05, indicating that the differences were statistically significant (Table 2). Both the PRF and NB groups showed alleviation of the pain of patients with PHN, with patients in the PRF group presenting long-term pain relief owing to the higher excellent, good, and overall response rates.

**Table 2:** Efficacy evaluation at postoperative 6 months in both groups.

**Group**	**Number of cases**	**Superior (case, %)**	**Good effect (case, %)**	**Fair effect (case, %)**	**Poor effect (case, %)**	**Overall response rate**
NB group	32	7,21.87%	10,31.25%	9,28.12%	6,18.75%	26,81.25%
PRF group	31	15,48.38%	7,22.58%	6,19.35%	3,3.22%	28,90.32%
*Z*-value		-2.055				
*P*-value		0.040				

NB, nerve block; PRF, pulsed radiofrequency.

### 3.6 Complications

No treatment-related complications, including local infection, local hematoma, and decreased visual acuity, were observed during treatment in both groups. Among the patients in the PRF group, one patient developed frontofacial scalp numbness, which was relieved after 1 week of intramuscular cobalamin, and three patients developed periocular ecchymosis, which was relieved after 1 week.

## 4 Discussion

O'Neill et al. (9) reported that approximately 10%−15% of herpes zoster cases involve the trigeminal nerve. Bouhassira et al. stated that PHN is a severe condition accompanied by local skin itching, hyperalgesia, and paresthesia, with pain lasting from months to decades, which considerably affects daily life and work (10). Traditional drugs and NBs are often unsatisfactory in treating PHN, which mainly manifested as intolerance to the side effects of the drug treatment or shorter maintenance time (2). Herein, PRF of the supraorbital nerve was used to treat the ophthalmic branch of PHN. PRF treatment could effectively relieve pain, improve sleep quality, and reduce the dose of pregabalin administered. All treatments in patients of the PRF group were ultrasound-guided, and the supraorbital nerve could innervate the frontofacial region through the supraorbital foramen or supraorbital notch out of the eyebrow arch, which is the anatomical basis for treating frontofacial PHN (Figure 2). Javier et al. used ultrasound-guided PRF of the infraorbital nerve to treat the second branch of PHN and reported that the structures around the nerve and blood vessels were observable under ultrasound, which facilitated avoiding of blood vessels (6); however, only one case was enrolled in this study, whereas studies on ultrasound-guided PRF of the supraorbital nerve are lacking. Using high-frequency ultrasound scanning, we found that the hyperechoic bone was interrupted by the supraorbital foramen or notch and observed the supraorbital artery, which provided a theoretical basis for our study.

Herein, we found differences between the VAS scores of the two groups at postoperative intervals of 1, 3, and 6 months and between the PSQI scores at a postoperative interval of 1 week. Furthermore, the PSQI and VAS scores in the PRF group were lower than those in the NB group, and the PRF group presented better pain control and sleep quality improvement than those in the NB group. The reason for this observation was that PRF regulation of the peripheral nerves has a good effect on neuralgia; Erdine et al. found that PRF can affect adenosine triphosphate metabolism and ion channel function in the sensory nerves, continuously and reversibly inhibit the occurrence of C-fiber-evoked potentials, and stimulate the central process to activate the brainstem descending inhibitory system to achieve endogenous analgesia (11); thus, it blocks pain conduction through related nerves. Liu et al. enrolled 32 patients with the first branch of PHN and used DSA-guided PRF of the trigeminal ganglion (5), and the results showed that the pain was considerably reduced in 30 patients after the PRF treatment. Compared with our study, the difference lay in the guidance mode and therapeutic target, which was operated under DSA, and the therapeutic position was the semilunar ganglion of the trigeminal nerve. Herein, we set the radiofrequency parameters for high voltage and long duration, with voltage being 70 V and time being 6 min at 40°C and 6 min at 42°C (12). The therapeutic parameters were 42°C, 2 Hz, 20 ms, and 8 min. Wan et al. used CT-guided puncture of the semilunar ganglion of the trigeminal nerve to treat the first branch of the postherpetic trigeminal nerve, with 60 patients in the long-term high-voltage group (40 V → 60 V to 100 V, 2 Hz, 20 mS, 900 S) and 60 patients in the standard group (40 V, 2 Hz, 20 mS, 120 S) for three cycles (13), and the results showed that the long-term high-voltage group had higher VAS score, 36-item short-form survey score, and pregabalin dose than those of the standard radiofrequency group; the adverse reactions were observed mainly in seven patients of the standard group and 11 patients of the long-term high-voltage group presenting with facial swelling. Herein, the supraorbital nerve was selected as the treatment target, and ultrasound guidance was employed for a long duration of 12 min at 70 V, which was one of the reasons for the high, excellent, and good rates (70.96%). Combined with clinical practice, our observation regarding CT-guided transforamen ovale puncture of the semilunar ganglion of the trigeminal nerve was as follows: The difficulties in regulating the semilunar ganglion of the trigeminal nerve were because of the need for guidance by a large-scale medical equipment, high technical requirements, long puncture path, swellings in easy-to-puncture blood vessels, decrease in the heart rate induced by the easy-to-occur vagal reflex when entering the foramen ovale, increased blood pressure, severe cardiac arrest, finding of the ophthalmic branch of the semilunar ganglion of the trigeminal nerve after entering the foramen ovale, increased radiation exposure due to repeated puncture scan, cerebrospinal fluid leakage, and the risk of a cerebral hemorrhage.

Liu et al. conducted a retrospective analysis of 32 patients with PHN (5); DSA was used to guide the percutaneous puncture of the semilunar ganglion of the trigeminal nerve, and 93.75% of patients achieved considerable pain relief, with only two patients returning to the hospital for treating recurrence; however, a control group was lacking in the study. Herein, six cases in the NB group and three cases in the PRF group had poor effects, which was higher than those in the aforementioned studies. The reasons for this observation were as follows: NB was effective but the duration was short and easy to repeat; the supraorbital nerve was one of the many branches of the ophthalmic branch, and other branches may not be regulated; and the regulation of semilunar ganglion of the trigeminal nerve may be superior compared to the peripheral nerve regulation. Regarding other treatment methods, Liu et al. (14) performed subcutaneous short-course peripheral nerve stimulation in 26 patients with the first branch of PHN in the subacute phase (30–90 days). The postoperative VAS score was considerably reduced, with a VAS score of >3 being reduced to < 4%, with an effective rate higher than that in the present study, and medication was also reduced; the reason was believed to be the long-term regulation of the diseased nerve (15), which was consistent with the results of long-term nerve regulation in the sciatic nerve injury rat model (5). Zhao et al. (16) reported a case study where cervical spinal cord stimulation combined with supraorbital subcutaneous electrical stimulation and transforamen ovale electrode placement achieved good clinical efficacy. Even though this was at the forefront of clinical practice, it was rarely carried out, and the study lacked large-sample data to confirm the efficacy and safety.

Gabrhelík et al. and Eghtesadi et al. reported that PRF is currently the more commonly used treatment for neuropathic pain (15, 17), especially for PHN (18, 19). PRF plays an important role in pain diagnosis and treatment and is an effective and convenient neuromodulation technique, that can efficiently alleviate pain and improve sleep quality when used in combination with NB. Simultaneously, ultrasound can be utilized to observe the real-time and dynamic adhesion and edema of the tissue around the supraorbital nerve to avoid important tissues such as the eyeball, supraorbital artery, and other structures, and allows treatment to be focused on the target tissues with lesions, optimization of the treatment plan, improvement of the efficacy, and providing of a new treatment plan and method for the ophthalmic branch of PHN. This study was a retrospective analysis; the patients lacked preintervention balance but were screened according to the inclusion criteria and followed up for 6 months, which is a relatively short follow-up time; thus, further study is needed for validating the long-term efficacy of the PHN treatment.

In conclusion, supraorbital nerve PRF can effectively control the pain of the first branch of the trigeminal nerve after herpes zoster, improve the quality of sleep, and reduce the dose of pregabalin; it is safe to be operated under ultrasound guidance, and it has little damage to the ocular tissues, vascular nerves, and so on. This method provides a new treatment option and treatment modality for the ocular branch of postherpetic trigeminal neuralgia. However, the first branch of postherpetic trigeminal neuralgia includes branches of the supraorbital nerve, the supratrochlear nerve, and the nasociliary nerve. This method treats most of the area innervated by the first branch of the trigeminal nerve, and the other innervated areas of the nerve still need to be further researched and explored for effective treatment modalities and methods. The parameters of PRF treatment of the first branch of trigeminal neuralgia after herpes zoster, such as the length of time of the pulse treatment and the amplitude of the voltage, need to be further explored and studied.

## Data availability statement

The original contributions presented in the study are included in the article/supplementary material, further inquiries can be directed to the corresponding author/s.

## Ethics statement

The studies involving humans were approved by the Affiliated Hospital of Southwest Medical University. The studies were conducted in accordance with the local legislation and institutional requirements. Written informed consent for participation was not required from the participants or the participants' legal guardians/next of kin in accordance with the local legislation and institutional requirements.

## Author contributions

FL: Writing—original draft, Writing—review & editing. GG: Formal analysis, Writing—review & editing. YZ: Data curation, Writing—review & editing. CO: Supervision, Writing—review & editing.

## References

[1] Li M Hu H Tong SX Tian J Zhang S Feng D. The therapeutic efficacy of pulsed radiofrequency alone versus a dexamethasone and pulsed radiofrequency combination in patients with trigeminal postherpetic neuralgia: a double-blind, randomized controlled trial. Pain physician. (2022) 25:E543–e549. 35793178

[2] Johnson RW Rice AS. Clinical practice. Postherpetic neuralgia. N Engl J Med. (2014) 371:1526–33. 10.1056/NEJMcp1403062 25317872

[3] Chua NH Vissers KC Sluijter ME. Pulsed radiofrequency treatment in interventional pain management: mechanisms and potential indications-a review. Acta neurochirurgica. (2011) 153:763–71. 10.1007/s00701-010-0881-5 21116663 PMC3059755

[4] Jia Y Chen Z Ren H Luo F. The effectiveness and safety of 42°C pulsed radiofrequency combined with 60°C continuous radiofrequency for refractory infraorbital neuralgia: a prospective study. Pain Phys. (2019) 22:E171–e179. 10.36076/ppj/2019.22.E171 31151340

[5] Liu DY Chen JS Fang ZZ Liu SY Wan L. Pulsed radiofrequency of the trigeminal ganglion for treating postherpetic neuralgia of the ophthalmic branch. Pain Res Manage. (2021) 2021:6638392. 10.1155/2021/9791801 34122683 PMC8189809

[6] Javier J Wilton J Galluccio F Allam AE. Pulsed radiofrequency for postherpetic trigeminal neuralgia: a case report. Cureus. (2022) 14:e28913. 10.7759/cureus.28913 36237778 PMC9547084

[7] Buysse DJ Reynolds CF 3rd Monk TH Berman SR Kupfer DJ. The Pittsburgh Sleep Quality Index: a new instrument for psychiatric practice and research. Psychiat Res. (1989) 28:193–213. 10.1016/0165-1781(89)90047-4 2748771

[8] Macnab I. Negative disc exploration. An analysis of the causes of nerve-root involvement in sixty-eight patients. J Bone Joint Surg. (1971) 53:891–903. 10.2106/00004623-197153050-00004 4326746

[9] O'Neill F Nurmikko T Sommer C. Other facial neuralgias. Cephalalgia Jn. (2017) 37:658–69. 10.1177/0333102417689995 28133989

[10] Bouhassira D Chassany O Gaillat J. Patient perspective on herpes zoster and its complications: an observational prospective study in patients aged over 50 years in general practice. Pain. (2012) 153:342–9. 10.1016/j.pain.2011.10.026 22138256

[11] Sluijter ME Teixeira A van Duijn B. Comment on: Erdine S et al.; Ultrastructural changes in axons following exposure to pulsed radiofrequency fields. Pain Pract. (2010) 10:262. 10.1111/j.1533-2500.2010.00387_1.x 20546522

[12] Zhang E Fei Y Xu L Huang B Yao M. Effect of repeated high-voltage long-duration pulsed radiofrequency on herpetic neuralgia. Pain physician. (2022) 25:E1047–e1055. 36288590

[13] Wan CF Song T. Comparison of two different pulsed radiofrequency modes for prevention of postherpetic neuralgia in elderly patients with acute/subacute trigeminal herpes zoster. Neuromodulation. (2022) 25:1364–71. 10.1111/ner.13457 34008278

[14] Liu DY Chen JS Lin CY Gong QJ Zhao Q Wan L. Subcutaneous peripheral nerve stimulation for treatment of acute/subacute herpes zoster-related trigeminal neuralgia: A retrospective research. Clin J Pain. (2021) 37:867–71. 10.1097/AJP.0000000000000981 34593674

[15] Gabrhelík T Michálek P Adamus M. Pulsed radiofrequency therapy versus greater occipital nerve block in the management of refractory cervicogenic headache - a pilot study. Prague Med Rep. (2011) 112:279–87. 10.1016/S1754-3207(11)70669-1 22142523

[16] Zhao L Song T. Case Report: Short-Term Spinal Cord Stimulation and Peripheral Nerve Stimulation for the Treatment of Trigeminal Postherpetic Neuralgia in Elderly Patients. Front Neurol. (2021) 12:713366. 10.3389/fneur.2021.713366 34413827 PMC8368125

[17] Eghtesadi M Leroux E Fournier-Gosselin MP Lespérance P Marchand L Pim H. Neurostimulation for refractory cervicogenic headache: a three-year retrospective study. Neuromodulation. (2018) 21:302–9. 10.1111/ner.12730 29178511

[18] Wang C Dou Z Yan M Wang B. Efficacy and safety of pulsed radiofrequency in herpes zoster related trigeminal neuralgia: a systematic review and meta-analysis. J Pain Res. (2023) 16:341–55. 10.2147/JPR.S396209 36756203 PMC9901482

[19] Jia Y Shen Y Meng L Wang T Luo F. Efficacy, safety, and predictors of response to pulsed radiofrequency therapy for acute zoster-related trigeminal neuralgia patients: a multicenter retrospective study. Pain Physi. (2022) 25:E523–e530. 35793176

